# Mercurious-vivus

**Published:** 1896-04

**Authors:** 


### HOMŒOPATHIC PHYSICIAN,

A MONTHLY JOURNAL OF

HOMŒOPATHIC MATERIA MEDICA AND CLINICAL MEDICINE.

"If our school ever gives up the strict inductive method of Hahnemann, we are lost, and deserve only to be mentioned as a caricature in the history of medicine."—CONSTANTINE HERING.

Vol. XVI. APRIL, 1896. No. 4.

### EDITORIAL.

MERCURIUS-VIVUS.—This remedy, like Arsenic, is one of the most important remedies in our materia medica. The old school practitioners say that our whole materia medica consists only of Mercury. They always charge us with giving Mercury. Sometimes they "hit it," because the physician may have given it at the time specified. But in what different condition is it prescribed from that supposed by his very sagacious (?) old school opponent! This condition is *so* different from that assumed by the aforesaid sagacious opponent that he is just as far wrong as if he had named some other drug. How can a potency, say the 30th or 200th of Mercurius-vivus, be compared with a four, six, or ten-grain dose of Calomel?

In the printed pathogenesis of Mercury we find the symptom "anxiety, apprehension, and restlessness, especially in the evening and at night with fear of losing one's mind and understanding."

The anxiety and restlessness are similar to Aconite. The fear of losing one's mind is similar to Calcarea-carb. The aggravation at night is one of the great characteristics of Mercury throughout its symptomatology. The indifference to everything sometimes goes on to unconsciousness. Mercurius has continuous moaning and groaning. Apis whines continually. If the reader will turn to the number for March, 1895, pages 106 and 107, and will there read the notes given upon whining, moaning, and groaning, he will have all that Dr. Lippe said upon the subject, and which he substantially repeated in his lecture on Mercury.

Mercury has hurried speech. This reminds us of Lachesis and Stramonium which have the characteristic, incessant talking. It is a characteristic of both remedies; it is the keynote of Lachesis.

Mercurius has bad effects from fright, and this reminds us of Lycopodium which has suppressed menses after fright. Mercurius has home-sickness which is similar to Capsicum and Phosphoric-acid.

Mercurius is useful in delirium and mental derangement of drunkards. So also are Lachesis and Arsenic.

Borax, like Mercury, has vertigo as if one were in a swing.

Mercurius has burning pain in the head, worse at night, better on sitting up. The burning pain is similar to Arsenic. The aggravation at night is similar to Aconite, and the amelioration on sitting up is similar to Belladonna.

Mercurius has inflammation of the brain with sensation as if the head were in a hoop. Belladonna and Sulphur have sensation of a tight band around the head and Apis has desire for a tight band around the head.

The Editor has some additional notes upon this symptom of a band or hoop around the head and other parts of the body. They are as follow:

Sensation as if the legs were tightly bandaged. Benzoic-acid.

Feeling about the chest as if bandaged, with irresistible desire to cough. Lobelia.

Sensation around different parts of a tight band. Sabadilla, Sulphur.

Sensitiveness about the stomach so that tight band or tight clothing cannot be borne. Spongia.

Tension about forehead as if from a band. Mercurius.

Sensation in forehead as if bound by a band. Mercurius-iodatus-ruber, Baptisia.

Sensation around the head above the ears as of a band, with soreness of scalp. Gelsemium.

Sensation of a band around the head from over-heated rooms; with coryza, congestion to the head, and nausea. Carbo-vegetabilis.

Headache with sensation of a band around the head from stooping. Spigelia.

Headache as if from a tense band reaching from nape of neck to the ears. Anacardium.

Headache as if from a hot iron bound around the head. Aconite.

Headache with amelioration from tying a tight band around the head. Hepar.

Sensation as if the skull were constricted by a tape. Nitric-acid.

Sensation around the ankles as if from a band or ligature. Aconite.

Sensation around the arms as from a band or ligature tied around them, causing the blood to rush into the hands, in evening. Nux-moschata.

Constriction about the abdomen as if from a tight band. Argentum-nitricum.

Sensation around lower part of stomach region as if surrounded by a tight band, or as if tightly laced. Ignatia.

Knees pain as if firmly bandaged when sitting. Aurum-metallicum.

Thighs feel as if bandaged. Aconite.

Legs feel as if tightly bandaged. Benzoic-acid.

